# RPLAD3: anomaly detection of blackhole, grayhole, and selective forwarding attacks in wireless sensor network-based Internet of Things

**DOI:** 10.7717/peerj-cs.1309

**Published:** 2023-03-27

**Authors:** Zainab Alansari, Nor Badrul Anuar, Amirrudin Kamsin, Mohammad Riyaz Belgaum

**Affiliations:** 1 Faculty of Computer Science and Information Technology, University of Malaya, Kuala Lumpur, Malaysia; 2 College of Computing and Information Sciences, University of Technology and Applied Sciences, Muscat, Sultanate of Oman; 3 Computer Science and Engineering, G. Pullaiah College of Engineering and Technology, Kurnool, India; 4 Faculty of Computing and Informatics, Multimedia University, Cyberjaya, Malaysia

**Keywords:** Wireless sensor network, Internet of Things, Routing attacks, Network layer attacks, RPL protocol, Grayhole attack, Blackhole attack, Selective forwarding attack, Anomaly detection, Internal attacks

## Abstract

Routing protocols transmit vast amounts of sensor data between the Wireless Sensor Network (WSN) and the Internet of Things (IoT) gateway. One of these routing protocols is Routing Protocol for Low Power and Lossy Networks (RPL). The Internet Engineering Task Force (IETF) defined RPL in March 2012 as a *de facto* distance-vector routing protocol for wireless communications with lower energy. Although RPL messages use a cryptographic algorithm for security protection, it does not help prevent internal attacks. These attacks drop some or all packets, such as blackhole or selective forwarding attacks, or change data packets, like grayhole attacks. The RPL protocol needs to be strengthened to address such an issue, as only a limited number of studies have been conducted on detecting internal attacks. Moreover, earlier research should have considered the mobility framework, a vital feature of the IoT. This article presents a novel lightweight system for anomaly detection of grayhole, blackhole, and selective forwarding attacks. The study aims to use a trust model in the RPL protocol, considering attack detection under mobility frameworks. The proposed system, anomaly detection of three RPL attacks (RPLAD3), is designed in four layers and starts operating immediately after the initial state of the network. The experiments demonstrated that RPLAD3 outperforms the RPL protocol when defeating attacks with high accuracy and a true positive ratio while lowering power and energy consumption. In addition, it significantly improves the packet delivery ratio and decreases the false positive ratio to zero.

## Introduction

A new networking paradigm known as the Internet of Things (IoT) has emerged due to advancements in wireless sensor networks (WSN), embedded systems, radio frequency identification, and smart sensor technology (Yousefpoor et al., 2021). The addition of low-power sensing devices is predicted to help numerous IoT applications, including smart grids, smart homes, smart healthcare, and smart cities. IoT analytics predicts there will be about 27 billion connected IoT devices by 2025 (Hasan, 2022). IoT is enabled by widespread low-power, lossy networks (LLNs) characterized by low throughput and high packet loss in their communication links. The resource-constrained devices (nodes) used in LLNs have low energy, memory, and computational power (Kim et al., 2017b). Routing protocols used in conventional networks must be revised for LLNs due to resource constraints, high packet loss, and insufficient network throughput. One of these routing protocols is RPL (Routing Protocol for Low-Power and Lossy Networks), which was standardized by a working group of the Internet Engineering Task Force (IETF) for Ipv6 as RFC 6550 in March 2012 (Hui & Vasseur, 2012). Many IoT applications use RPL because it can provide LLNs with energy-efficient routing.

Due to the open, resource-constrained, self-organizing, and self-healing characteristics of RPL-based networks, which make them susceptible to various threats, users’ security and privacy are at risk. The primary objective of the RPL is not to defend systems against internal attacks (Kim et al., 2017b). A node typically behaves abnormally in an internal routing attack like a grayhole (Sidhu & Sachdeva, 2020), blackhole (Pawar & Jagadeesan, 2021), or selective forwarding (Lal & Prathap, 2021). These attacks are the most typical RPL attacks that drop some or all forwarded packets. The RPL cryptographic algorithm used to defend against external threats are ineffective when nodes are authenticated internally (Shim, 2016).

Maintaining the security of the collected data in RPL networks is essential for preserving the integrity of trust values. Moreover, the dynamic characteristic of RPL routing topology, which presents the region for receiving nodes from attackers, is a significant concern (Liu, Peng & Zhong, 2021). Besides, in addition to the high overheads like power and memory, most mechanisms do not consider the mobility of nodes and networks. Existing approaches depend on static trust frameworks and ignore dynamic and mobile LLN nodes (Wang et al., 2017).

Considering the above problems with RPL, trust-based security is strongly suggested for preventing internal attacks. It is necessary to use trust model security to keep track of a node’s packet-forwarding behavior. It can help detection of malicious entities in a network to ensure secure and continuous communications (Alansari et al., 2019). However, most of the current trust model mechanisms should use memory, bandwidth, and power resources more. A mechanism that uses the least energy at the node level must be developed to extend the RPL network lifetime. The current trust model’s complex computations will exhaust the sensor node’s resources. Furthermore, in a trust model mechanism that controls messages and sense data, trust-related information is multi-hop forwarded to the root or transferred between nodes, resulting in extra traffic and congestion (Lal & Prathap, 2021). High message overhead in both scenarios can affect network performance resulting in delays and packet loss. Thus, trust parameters and strategies for low-power, resource-constrained, and lossy networks are needed (Alansari et al., 2022).

This study aims to use a trust model in RPL protocol to increase network performance and lifetime while reaching high detection accuracy. The proposed system with the code name RPLAD3 is a trust model internal routing attack detection system to secure RPL protocol in a distributed IoT. It detects and prevents three routing attacks: grayhole, blackhole, and selective forwarding. RPLAD3 calculated the trustworthiness of the preferred parent node by counting its positive and negative behavior according to forwarded packets. The data packets are separated from control packets to differentiate between different attacks. The proposed system assesses two different thresholds to ensure detection accuracy. The trust thresholds are adaptive and can change according to the network’s security policy. The main aims of this study are as follows:

1. To investigate the limitations of routing attack detection systems in RPL Based IoT.
2. To use a trust model to propose anomaly detection for RPL-based routing attacks.
3. To evaluate the network performance using the new system by measuring the detection system accuracy, adaptivity, scalability, and mobility.

The following sections elaborate on this study’s overall flow, which aligns with the above goals. The rest of the article is as follows: “Literature Review” discussed the limitations of RPL protocol and current studies on three internal routing attacks: blackhole, grayhole, and selective forwarding. “Design and Methods” elaborates on the proposed system architecture and frameworks, while “Results” and “Discussion” present the simulation results and discussion following a comparison analysis of RPLAD3 with standard RPL protocol. Finally, “Conclusions” supplies the overall conclusion of the study.

## Literature review

**Figure 1:**
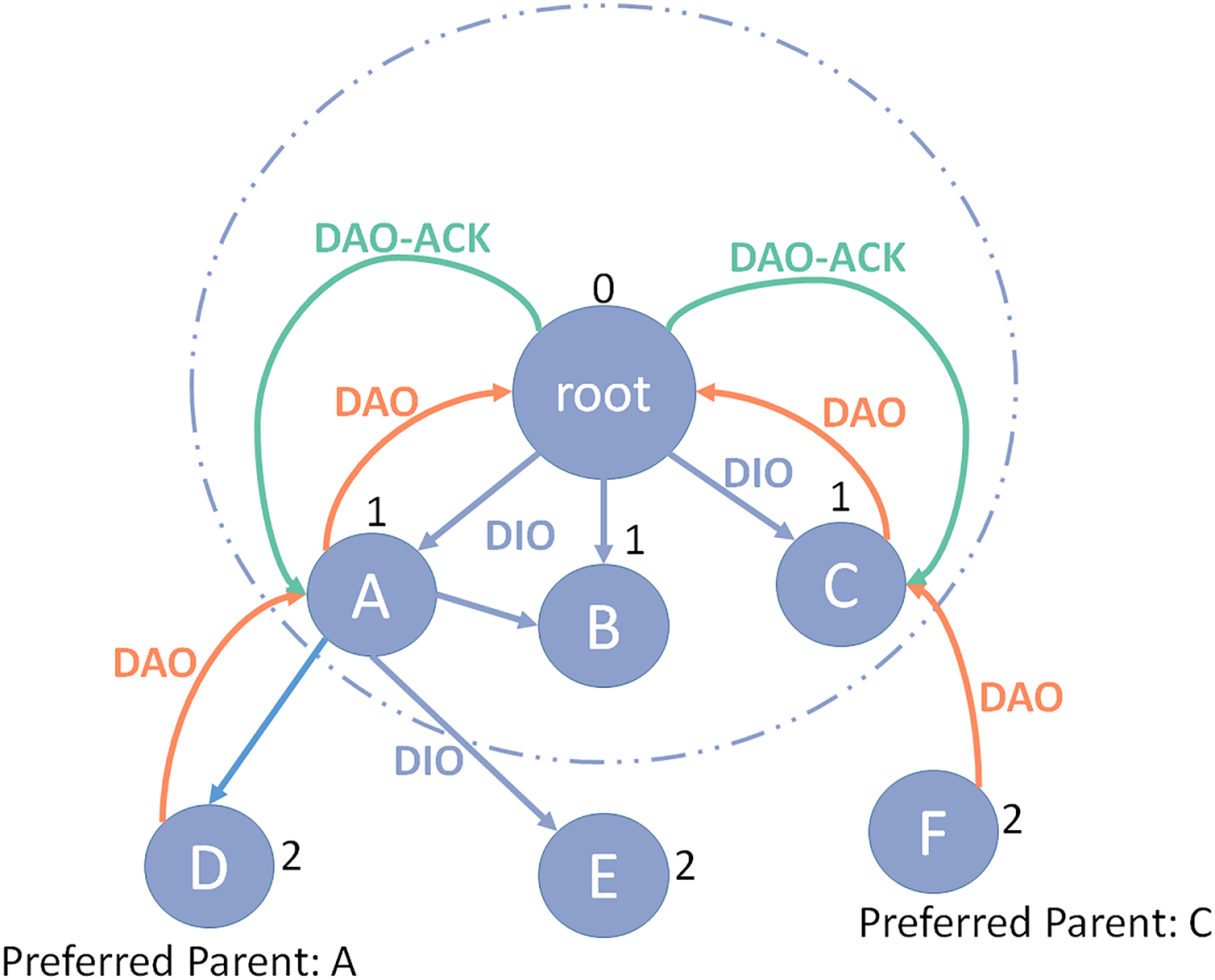
RPL protocol structure.

RPL, which stands for IPv6 Routing Protocol for Low Power and Lossy Networks (LLNs), is a distance vector routing protocol and can be routed to various mechanisms of the different Network layers. RPL acts by the topological concept of Destination-Oriented Directed Acyclic Graph (DODAG) (Alansari et al., 2022). DODAG means a directional graph without a destination and has a tree configuration that delimits the default paths of the network. Notice that DODAG is more than a regular tree structure, as in DODAG, each node can consume more than one parent, while in a traditional tree structure, each node has only one parent. The construction of DODAG consists of two steps, which will be discussed below (Kim et al., 2017a). Figure 1 illustrates the RPL protocol structure.

### Upward route

The directed acyclic graph (DAG) information object (DIO) message that the DODAG route sends to indicate the graph ID and its rank is used to construct DODAG. It allows other nodes to recognize their position within the network. DIO message will be sent to other nodes if new nodes want to join the network. By receiving the DIO’s message, it adds the address of the DIO message sender to its parent list and calculates their rank with the help of the objective function (OF). The rank of each node should be higher than the rank of all its parents. Furthermore, it updates the DIO packet with its ran and redistributes it all again. This procedure is recapped until all the nodes in the network receive their ranking. Each node should select a node from its parents as its preferred parent to direct data packets to the sink. When a node joins the DODAG graph, if it senses a DIO message, it can process it in three ways:

- Drop the DIO packet under some RPL terms.
- Route the message to preserve its location in the network.
- Expands its location by getting a lessened rating inside DODAG.

Whenever a node reduces its rank, it must remove all the parental members who rank less than their ranking from their parent list to stop the loop created in the network. After the end of this phase, each node has a default path to the root and can send its data packets to the root (Oliveira & Vazão, 2016).

### Downward route

If the flag type is a non-zero performance in the DIO message, the downward route should be supported and kept from root to nodes. Each node must send a destination advertisement object (DAO) message to its parent to determine the downward route information. The DAO packets transmit node addresses that cross paths along the upward route when they move from the nodes to the root, and the entire path between the root and the nodes is created. This message can be acknowledged by the destination using DAO’s acknowledgment message. RPL has two functional models for maintaining a downward route:

- Storage mode, in which the parent receiving the DAO packet can save the content of the DAO message before sending the packet to its members. In this case, each parent node stores the address of all its child nodes.
- Nonstorage mode, in which a direct DAO message is sent to the root of DODAG. Thus, the source nodes reject the DAO’s message storage, only store their addresses in the route, and direct it to their chosen parent. As a result, no parent maintains the address of its child’s nodes, and the only root that receives all of the DAO’s packets can store the entire downward route (Kim et al., 2017b).

Since traditional Internet routing protocols are insufficient for resource-constrained IoT devices and routing techniques perform poorly in the IoT, several solutions have been proposed to manage routing. One of these standards, the RPL, was developed from scratch to address the routing requirements of IoT networks and minimize resource consumption along the route (Hui & Vasseur, 2012). RPL has its security methods primarily *via* the following three security modes:

- Unsecured mode: RPL’s default setting when link-layer security is needed. The RPL control messages are not subject to security controls in this mode.
- Preinstalled mode: Symmetric preinstalled keys are manually preconfigured on the nodes. These keys are used to manage and produce secure copies of RPL control messages when a node joins the DODAG and for keeping them. This possibility is recommended when secure routing is necessary with many limited devices.
- Authenticated mode: Nodes with routing capabilities must use the preinstalled keys to get another key from the authentication authority. In this mode, nodes that wish to join the DODAG as leaf nodes use the preinstalled keys to join. The implementation will decide how the authority verifies the nodes and how the keys are shared.

Furthermore, RPL employs AES/CCM (the Advanced Encryption Standard in the “Counter with Cipher-block chaining Message authentication code” mode) with a 128-bit key to create 32-bit and 64-bit Message Authentication Codes to keep the confidentiality, integrity, and authenticity of these communications (MAC). These MACs are used to guarantee the communications’ integrity. To ensure the privacy and validity of the communications, RPL also employs RSA with SHA-256 for the optional 2,048- and 3,072-bit digital signatures (Raoof, Matrawy & Lung, 2018).

However, RPL cryptographic algorithm and authentication key generation cannot detect internal attacks. Selective forwarding, grayhole, and blackhole attacks are typical internal routing attacks. A blackhole attack creates a “black hole” in the network by having malicious nodes delete all packets they receive rather than forwarding them, leading to a Denial of Service (DoS) (Pawar & Jagadeesan, 2021). A more advanced attack is the selective forwarding attack which sends packets from a few protocols while dropping the rest, for example, only sending ICMPv6 and RPL control packets while deleting all other packets (Lal & Prathap, 2021). Grayhole attack changes in the packet before forwarding them (Sidhu & Sachdeva, 2020). These three attacks differ primarily in their goals: selective forwarding and grayhole attacks aim to significantly disrupt routing, while the blackhole attack desires to impose a DoS on the network. RPL’s self-healing capabilities cannot prevent or reduce these attacks since malicious nodes often transfer control messages from RPL and participate in forming and maintaining DODAGs like any other valid node.

The literature on routing attack detection is currently more constrained and needs to consider new protocols like RPL and their parameters to detect and prevent attacks. Internal attack detections in RPL networks have received little attention, but a limited detection system may be found using a mobility framework. The limited node’s capacity further complicates finding the right node to support the resource constraint detection approach. Patel & Jinwala (2022) developed an internal routing attack detection system for RPL protocol which on reputation calculation of node behaviour. Their behavior can detect selective forwarding attacks by checking actual packet loss to estimate the average loss. However, this system is ineffective in large-scale routing and does not support mobility frameworks.

Farooq et al. (2022) proposed MMTM-RPL system to detect three types of internal attacks based on RPL protocol. They integrated the trust model into RPL and developed a multi-mobile agent framework that supports mobility. Thus, their system consumes high energy and end-to-end delay due to the layer-based architecture. Similarly, Hashemi & Shams Aliee (2019) developed a mobility RPL based trust model to detect blackhole, sybil, and rank attacks. The significance of their system is the improvement in terms of an average number of parent changes; however, it consumes high energy and memory overhead. Another mobility system based on RPL was proposed by Medjek et al. (2021) to mitigate DIS attacks by modification of some RPL standard codes in the cooja simulator. It supports both static and dynamic RPL networks; however, it has high computational costs due to the use of added hardware omnidirectional. Table 1 is a comparison table to discuss each method and their strength and evaluation metrics.

**Table 1:** Comparison analysis.

Citation	Attack type	Detection method	Performance evaluation metric	Strengths
Bilgin & Baktir (2019)	Blackhole	Symmetric key cryptographic algorithm, AES, Signature Based	End-to-end delay, PDR	Does not cause extra communication delay
Nosratian, Moradkhani & Tavakoli (2021)	Blackhole	Fuzzy based, data mining, Genetic Algorithm (GA), teaching learning-based optimization (TLBO)	Computational cost	Least cost for the target performance
Pawar & Jagadeesan (2021)	Blackhole	Deep learning	TDR	Improves the detection probability
Saravanakumar et al. (2022)	Blackhole	Encryption method, artificial deep neural networks	PLR, Computation overhead, throughput, End-to-end delay	Achieve higher data delivery with a minimum delay
Sunder & Shanmugam (2019)	Blackhole	JDICA technique	False alarm rate, End-to-end delay, Detection accuracy, PDR, TDR, Energy consumption, Detection time	Greater accuracy, Increasing the packet delivery ratio, Reducing delay
Yadav & Mishra (2020)	Blackhole	Blockchain	End-to-end delay, PDR, Throughput	Successful identification and occurrence of malicious nodes
Chinnaraju & Nithyanandam (2022)	Grayhole	Neighbour based, Threshold Based	PDR, Network performance	Instead of blocking the entire host, it specifically eliminates the malicious nodes
Terence & Purushothaman (2019)	Grayhole Blackhole	Behaviour based	End-to-end delay, Throughput, FPR, PDR, FNR	Improve packet delivery ratio, Throughput, and end to end delay, Lesser false positive and false negative rate
Alqahtani et al. (2019)	Grayhole, Blackhole	Genetic algorithm, extreme gradient boosting (XGBoot) classifier	TDR	High detection rate
Derhab et al. (2020)	Selective forwarding	Neighbour based	Detection accuracy, Energy consumption	Defending against upstream node attack
Devi & Ganesan (2019)	Selective forwarding	Trust based, Watchdog	PLR	Pay attention to consecutive packet dropping
Fang et al. (2020)	Selective forwarding	Trust based	DTR, Energy consumption	Prevent the appearance of network holes, Balance the network load, Promote the survivability of the network
Fu et al. (2020)	Selective forwarding	Data Clustering Algorithm (DCA)	FDR, Energy consumption, MDR	Low missed detection rate, False detection rate, Low energy consumption
Ji (2018)	Selective forwarding	Threshold based	DTR, Resource consumption, Communication overhead	Saves communication resources
Kaur et al. (2019)	Selective forwarding	Threshold based	PLR, Throughput	Improvement in terms of dead nodes, Throughput, and packet loss
Lal & Prathap (2021)	Selective forwarding	Provenance based technique	Throughput	Assuring data trustworthiness
Liu & Wu (2021)	Selective forwarding	Cluster based, voting decision method	FDR, Energy consumption, MDR	Low FDR, Low MDR, Negligible energy consumption
Mehetre, Roslin & Wagh (2019)	Selective forwarding blackhole	Trust based, Cuckoo search algorithm	Network lifetime and performance	Prolong the network lifetime and the probability of secure routing path in the network
Pu & Lim (2018)	Selective forwarding	Timeout and hop by hop retransmission techniques	PLR, FDR, PDR, TDR, Energy Consumption	Improve the detection rate and PDR, reduce the energy consumption, false detection rate, and successful drop rate
Singh & Saini (2021)	Selective forwarding	Learning based	DTR, Efficiency	Better data transmission
Zhang & Zhang (2019)	Selective forwarding	Watchdog	False alarm rate, FDR, Detection accuracy, Energy consumption	Reduces the false detection rate by 25% and improves the detection accuracy by 10%

Thus, a resource-constrained internal attack detection system that supports a mobility framework and effectively detects attacks is required to resolve the identified issues.

## Design and methods

IoT and its applications have benefits for various industries to improve the safety of employees and manufactured products. IoT helps industries track the conditions and achieve better results (Alansari et al., 2020). The safety of employees is also of immense importance in industries such as mining, oil and gas, chemical, and power plants; Therefore, the existence of smart systems and sensors in these places helps to prevent accidents and significant industrial risks.

Today, IoT covers a large part of human life, and the adoption of this technology has doubled its importance. Because hackers and advanced persistent threats can act quickly and stealthily, security teams must provide up-to-date and accurate information to ensure automatic and precise defense tuning (Alansari et al., 2018). By providing security on the IoT, humanity will gain peace of mind and a sense of security in the age of the Internet and technology.

**Figure 2:**
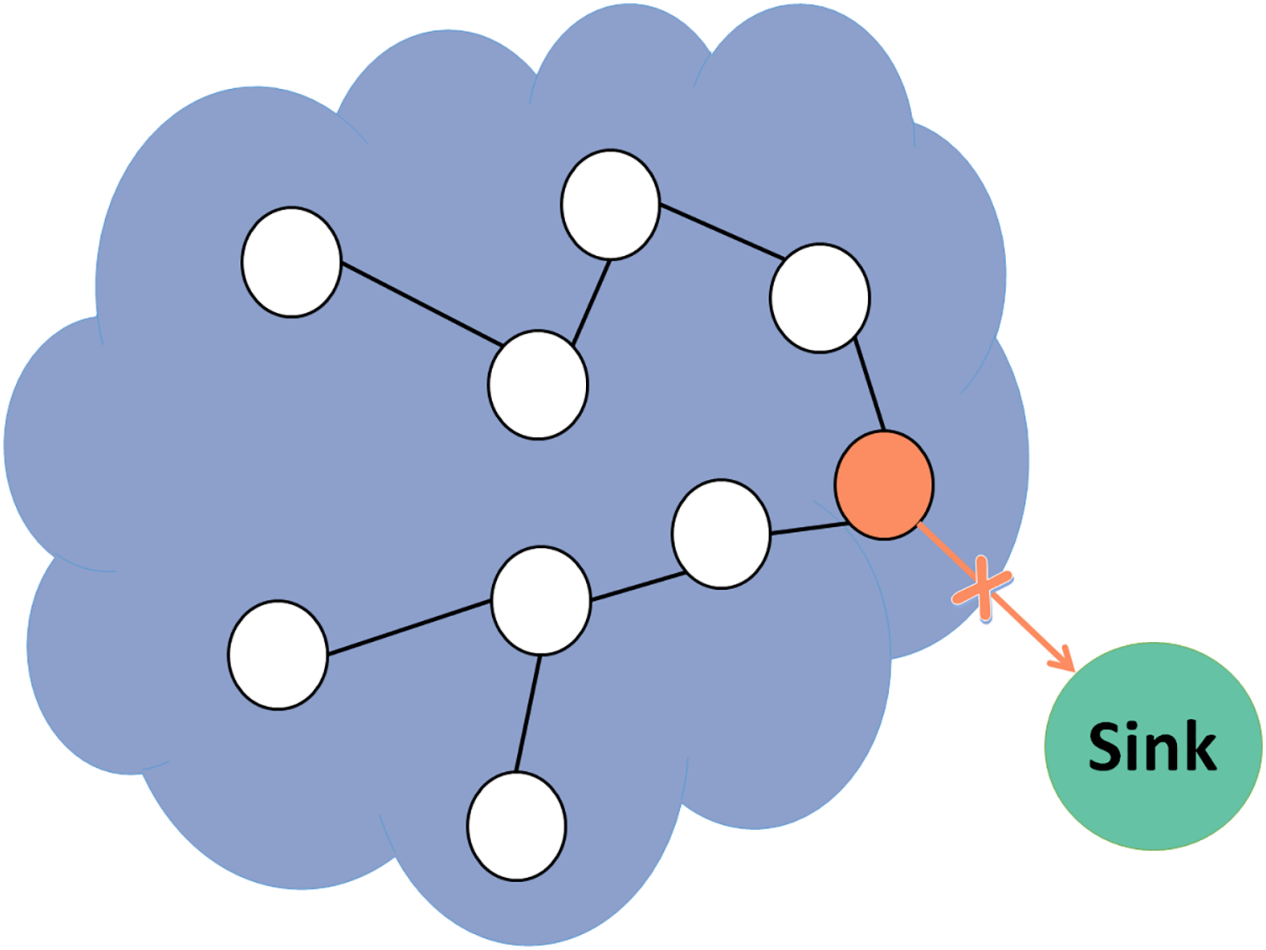
Blackhole attack.

This study aims to utilize a trust model in RPL protocol to increase network performance and lifetime while attaining high detection accuracy. This article presents a novel lightweight system for anomaly detection of three internal routing attacks: blackhole, grayhole, and selective forwarding attacks. In the blackhole attack shown in Fig. 2, the malicious node drops all forwarded packets.

**Figure 3:**
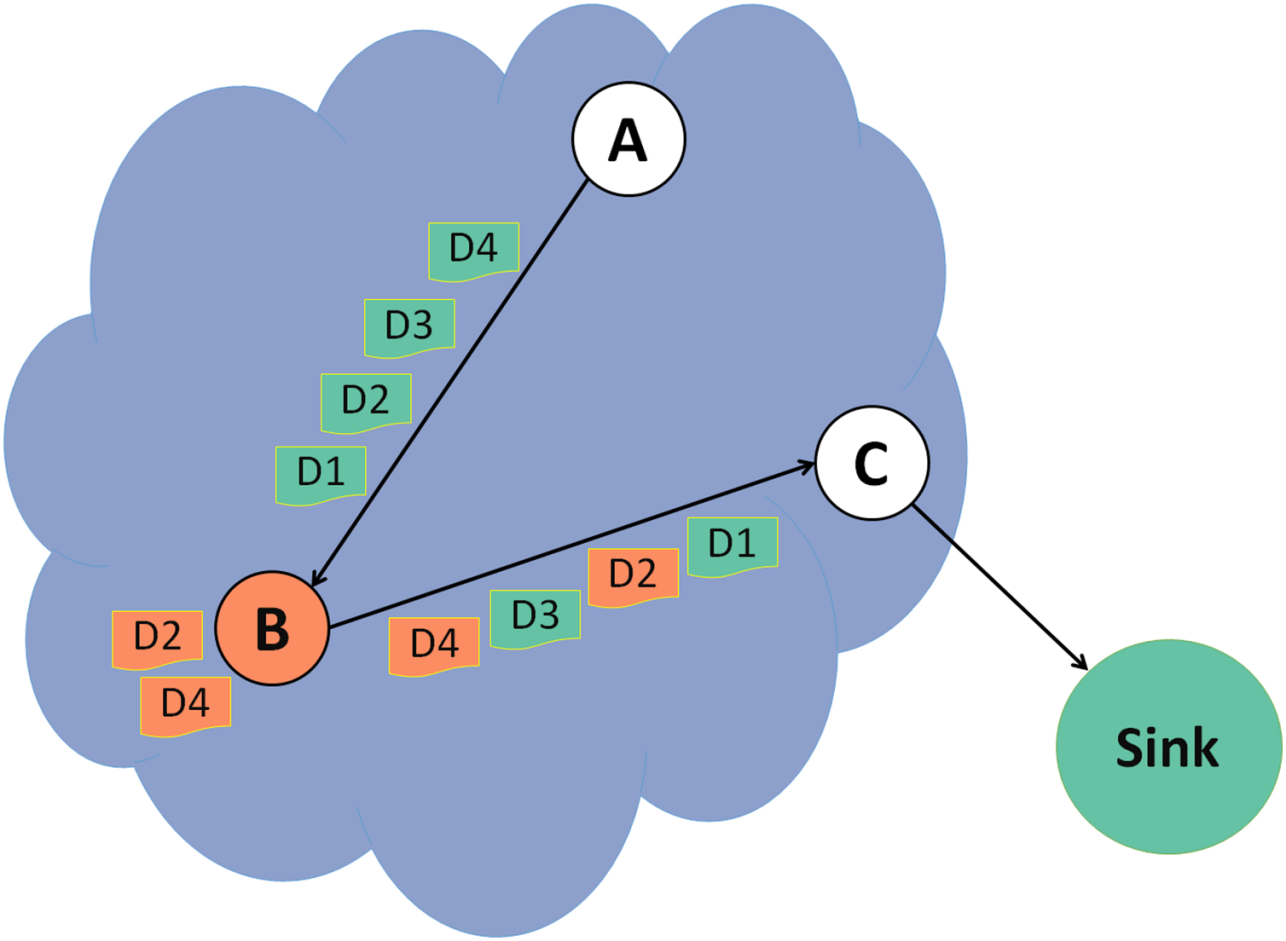
Grayhole attack.

In Fig. 3, node A forwards control packets and data packets to node B, a malicious node. Node B forwards control packets and changes some data packets before forwarding them to the next node. In this example, the changes are done on data packets 2 and 4.

**Figure 4:**
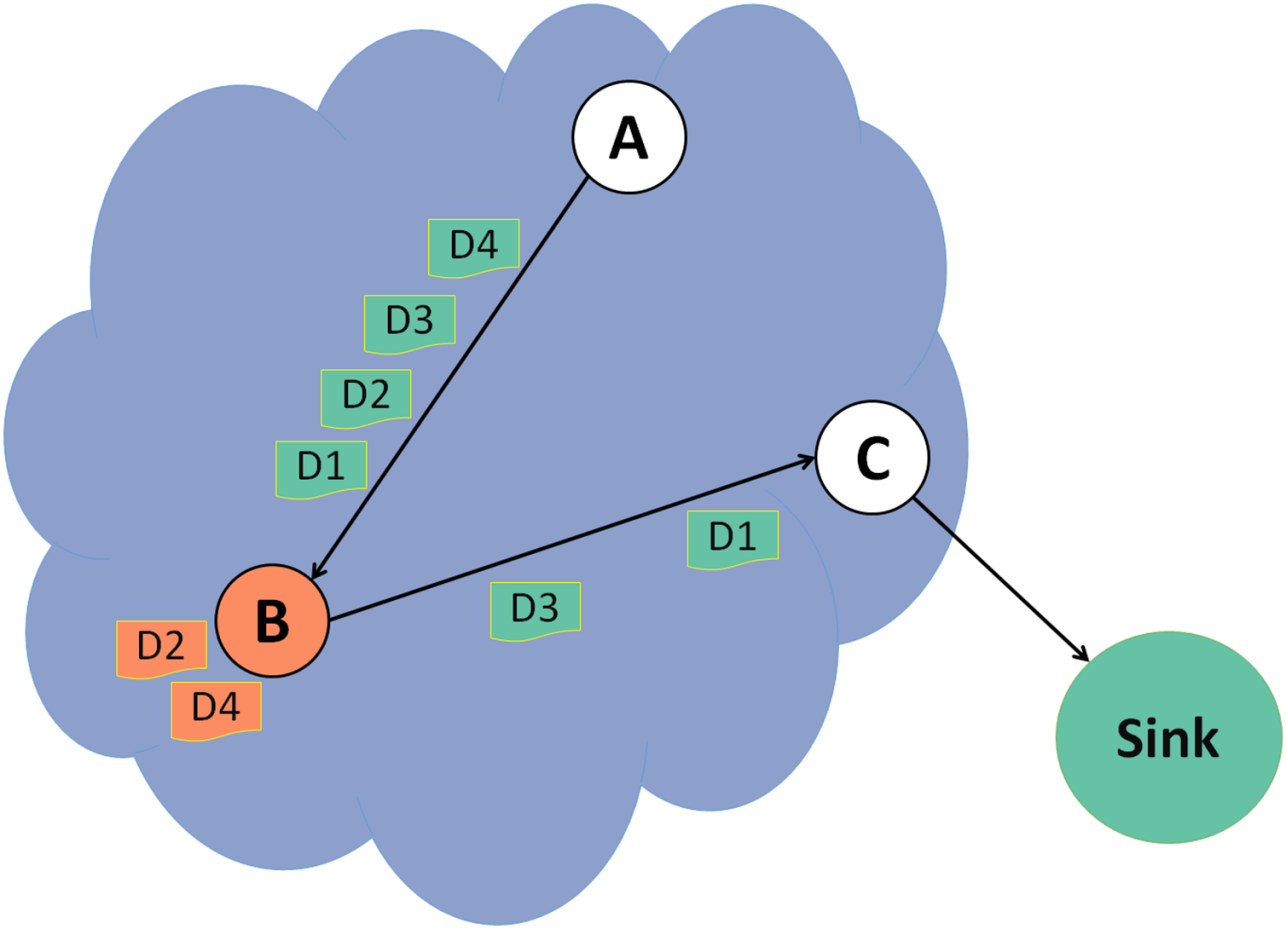
Selective forwarding attack.

In a selective forwarding attack illustrated in Fig. 4, a malicious node B drops some data packets and forwards the rest. Detecting this attack is more challenging as the malicious node usually forward all the control packets to keep the DODAG and will drop only some of the data packets. In this example, data packets 2 and 4 are dropped, and the rest are forwarded successfully to the destination.

The proposed RPLAD3 is a trust model routing attack detection system to secure the RPL protocol in a distributed WSN-based IoT. It detects and prevents three routing attacks: grayhole, blackhole, and selective forwarding. RPLAD3 calculated the trustworthiness of the preferred parent node by counting its positive and negative behavior based on forwarded packets. The data packets are separated from control packets to differentiate between different attacks.

The proposed system assesses two different thresholds to ensure detection accuracy. The first threshold is the trust threshold (α), which is based on the trust model and has an initial value of 0.5 minus the PLR. Prior to the actual startup, the administrator must run the network for about a week to calculate the PLR value. The trust threshold is adaptive and can change according to the network’s security policy. The second trust is the trust window which counts the number of times a node behaves negatively. It is also adjustable by the network administrator based on its needs and conditions. This is to provide a chance for nodes with temporary issues such as hardware failure, network congestion, or nodes that are greedy to save their resources and energy.

The novelty of the proposed system is as per below:

- It is applicable and resource-constrained in a distributed WSN based IoT as it is lightweight and utilizes a simple framework.
- It is adaptive and outperforms even in large networks, providing scalable algorithms for trust calculation and isolation of attacker nodes.
- It detects and isolates malicious nodes and provides a secure route for sensor nodes.
- It is developed for networks that require secure control and packet exchange with high levels of availability and integrity.
- It is compelling and reliable due to its remarkably high detection accuracy, low false positive rate near zero, and incredibly high true positive rate of almost one.
- It is designed in four interconnected layers tohave a full detection capability while detecting attacks.
- It operates within the mobility framework and can fulfill current industrial desires formed by mobile technologies.
- It is emulated on sensor nodes with complete setups and routing protocols for all layers, including the physical layer, to be capable of operating with actual sensor nodes.

### RPLAD3 architecture

The proposed system architecture is made up of four interconnected layers. Since these layers are connected, each can call the next layer whenever required. Different sublayers are incorporated in each layer which is discussed in detail in the following sections. The four layers of RPLAD3 are as follows:

- Layer one: information gathering.
- Layer two: trust calculation.
- Layer three: decision making
- Layer four: backup and restore.

**Figure 5:**
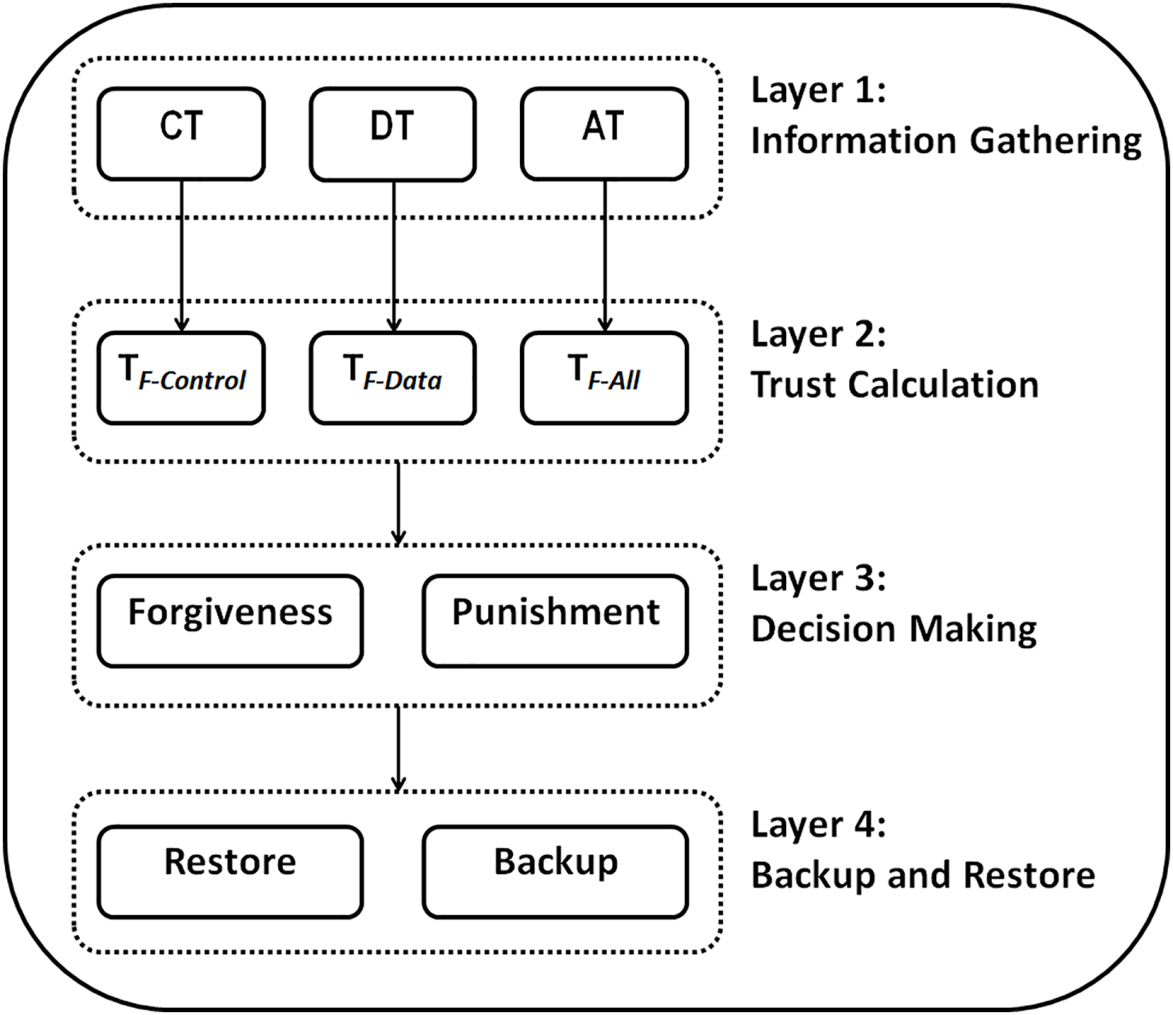
RPLAD3 architecture.

Attacks are detected in a distributed network without the intervention of a central network entity during the deployment of the proposed trust model, which is applied to each network node separately. Figure 5 illustrates the proposed method architecture.

All the required information for the trust calculation layer is collected in the information gathering layer. Additionally, in the trust calculation layer, the trust values about the preferred parent node are calculated using the data obtained from the previous layer. Furthermore, decisions regarding the trust in the preferred parent node are made in the decision-making layer using values obtained from layer two. The root will store the node’s crucial data during the backup and restore layer to access it in case of an error. One of the proposed system’s novelties is that it considers the backup and storage layer, which is uncommon in RPL-based systems.

The proposed method starts functioning immediately after the initial state of the network. At the start of the method, the information gathering layer monitors and collects information about the neighboring nodes. The trust calculation layer is called by layer one if required, and the other layers may also be called by the previous layer if essential. In the following sections, each layer will be discussed separately.

### Layer one: information gathering

Layer one collects three types of information from all immediate neighbours through three sublayers. Algorithm 1 displays step by step how layer one functions.

- CT: information related to control packets.
- DT: information related to data packets.
- AT: information related to all packets (Control and Data Packets).

**Algorithm 1:** Layer one

**Input**: Sent_Packet (DATA or DAO or DAO-ACK (in non-storing mode))
**Input**: Destination of the packet: j
**Begin**
**If** (watchdog(Sent_Packet,j) = = 1) //Algorithm 2
Switch Sent_Packet.Type
Case Data:
$p_j^{Data} + \; p_j^{Data} + 1$
Case Control:
$p_j^{Control} + \; p_j^{Control} + 1$
**Else**
Switch Sent_Packet.Type
Case Data:
${\rm \; \; \; \; \; \; \; \; \; \; \; \; \; \; \; }n_j^{Data} + \; n_j^{Data} + 1$
Case Control:
${\rm \; \; \; \; \; \; \; \; \; \; \; \; \; \; \; }n_j^{Control} + \; n_j^{Control} + 1$
**End If**
**If** ( $p_j^{Data} + p_j^{Control} + n_j^{Data} + n_j^{Control}$)> $\; C_j^{Data}$)
//Trigger DT sub_module from Trust Calculation layer
**End If**
**If** ( $p_j^{Data} + p_j^{Control} + n_j^{Data} + n_j^{Control}$)> $\; C_j^{Control}$)
//Trigger CT sub_module from Trust Calculation layer
**End If**
**If** ( $p_j^{Data} + p_j^{Control} + n_j^{Data} + n_j^{Control}$)> $\; C_j^{All}$)
//Trigger AT sub_module from Trust Calculation layer
**End If**
**End.**

#### AT (information about all packets)

Here we will explain the information gathering procedure for all control and data packets from a neighbor node. Let us assume that the calculations are done in node i and node j is the immediate neighbours of node i. $p_j^{All}$ is the overall number of positive behaviors observed from node j, and $n_j^{All}$ is the overall number of negative behaviors observed from node j. These values are calculated for all control and data packets. Node i goes into the promiscuous mode of its network card after sending any data packet or DAO or DAO-ACK packet to node j. This part uses the Watchdog algorithm, as seen in Algorithm 2. Promiscuous mode is a Wireless Network Interface Controller (WNIC) setting instructing the controller to pass all traffic in the data link layer. In normal mode in the network layer, nodes can only observe the forwarded packets with their MAC address. In contrast, in promiscuous mode, nodes in the network layer can observe all forwarded packets even if they do not have their MAC address. This will not increase energy consumption as RPLAD3 only consumes a small amount of energy except for the initial energy required to transmit a packet. Furthermore, the attacker node will be blocked after detection, limiting the number of times this scenario is repeated to two or three.

**Algorithm 2:** Watchdog

**Input:** Sent_Packet (DATA or DAO or DAO-ACK (in non-storing mode))
**Input:** One-hop Neighbor that sent_packet is forwarded to: j
**Begin**
$F \leftarrow 0$
Set_watchdog_timer ()
**While** (!expired(watchdog_timer))
//listen to all packets in promiscuous mode
** For** each received packet p from j
** If** (p = sent_packet)
${\rm \; \; \; \; \; \; \; \; \; \; \; \; \; \; \; }F \leftarrow 1$
Break
** End If**
** End For**
**Return F**
**End While**
**End.**

The watchdog algorithm’s timer, t, is based on the maximum one-hop delay between two neighboring nodes. According to our estimate of 1 s, parents have that much time to deliver and forward the packet. Based on our simulations, we determined the time to be one since we had never experienced longer packet delays. If it finds that node j forwards that packet unchanged, it will increase the number of positive behaviors of node j as per Eq. (1):

(1) $$p_j^{All\,\left( {new} \right)} = p_j^{All\,\left( {old} \right)} + 1$$

Otherwise, it increases the number of negative behaviors of the node j as per Eq. (2):

(2) $$n_j^{All\,\left( {new} \right)} = n_j^{All\,\left( {old} \right)} + 1$$

It then stores these two numbers in one byte each, along with the information stored in the protocol for parent node j. If $C_j^{All}{\rm \; }$ packets are routed to node j, the trust threshold for the successful forwarding of all packets (T_F-All_) under Trust calculation layer for node j will be called.

#### CT (information about control packets)

Here we will explain the information gathering procedure for control packets from a parent node. If DAO or DAO-ACK control packets (the only multicast control packets in RPL) are forwarded to parent node j, the positive $p_j^{Control}{\rm \; }$ or negative $p_j^{Control}{\rm \; }$ behaviour of node j about control packets will be calculated. If node j forwards the DAO or DAO-ACK control packets without any changes, Eq. (3) will take place:

(3) $$p_j^{Control\,\left( {new} \right)} = p_j^{Control\,\left( {old} \right)} + 1$$

Otherwise, Eq. (4) will be used:

(4) $$n_j^{Control\,\left( {new} \right)} = n_j^{Control\,\left( {old} \right)} + 1$$

If $C_j^{Control}{\rm \; }$ packets are routed to Node j, the trust threshold for successfully forwarding packets (T_F-Control_) under the trust calculation layer for node j will be called.

#### DT (information about data packets)

The positive behaviour about forwarding data packets by node j is $p_j^{Data}$ and the negative behaviour about forwarding data packets by node j is $n_j^{Data}$. These values are simply calculated from the difference between the behaviors of all packets and the behaviors of the control packets as shown in Eqs. (5) and (6):

(5) $$p_j^{Data} = p_j^{All} - n_j^{Control}$$

(6) $$n_j^{Data} = n_j^{All} - p_j^{Control}

These values are used to detect selective forwarding attacks that selectively forward the control packets while discarding the data packets. If $C_j^{Data}{\rm \; }$ packets are routed to node j, the trust threshold for successfully forwarding packets (T_F-Data_) under the trust calculation layer for node j will be called.

### Layer two: trust calculation

In this section, we describe the process used to calculate trust. The three sublayers of this layer are the calculations of the trust that each node obtains about its parent node.

The trust threshold is measured as a decimal value between zero and one. The initial value is 0.5-PLR (packet loss rate), which expresses a neutral point of view toward the nodes at the initial state of the network. According to the information obtained from the parent node in the information gathering layer, trust can increase or decrease over time.

Trust is calculated for the successful forwarding of packets using the obtained information from layer one about a parent node’s positive and negative behaviour. For this purpose, first the trust for all the successful forwarded packets will be calculated as *TF-All*. Then the trust for the successful forwarding of control packets *TF-Control* is calculated and then the trust for the successful forwarding of data packets *TF-Data* is calculated. Finally, it obtains the minimum of these three values as the final value for trust_._

#### TF-ALL

Node i calculates trust for the successful forwarding of all packets by node j as per Eq. (7):

(7) $$T_{All}^j = \displaystyle{{p_j^{All} + 1} \over {p_j^{All} + {\rm \; }n_j^{All} + 2}}$$

#### TF-Control

Node i calculates the trust for successful forwarding of control packets by node j as per Eq. (8):

(8) $$T_{Control}^j = \displaystyle{{p_j^{Control} + 1} \over {p_j^{Control} + {\rm \; }n_j^{Control} + 2}}$$

#### TF-Data

Node i calculates the trust for successful forwarding of data packets by node j as per Eq. (9):

(9) $$T_{Data}^j = \displaystyle{{p_j^{Data} + 1} \over {p_j^{Data} + {\rm \; }n_j^{Data} + 2}}$$

It can be observed that the trust value is 0.5 at the initial state of the network when no positive or negative behaviour of node j is found, which defines the reason that the trust threshold is 0.5-PLR. If the network administrator is required to change the trust threshold at the initial state of the network, the initial value of *p*_*j*_ or *n*_*j*_ must change.

Finally, the minimum of TF-ALL, TF-Control and TF-Data is calculated to obtain final trust value as per Eq. (10):

(10) $$T_F^j = {\rm MIN}\left( {T_{All}^j,T_{Control}^j,T_{Data}^j{\rm \; }} \right)$$

Whenever any node in a network forwards any DAO, DAO-ACK, or data packet to its parent, it will wait for the t second to search in the watchdog algorithm to observe whether the parent forwarded the packet. The t second is calculated based on the one hop delay in the network. The network administrator using RPLAD3 must calculate one hop delay by running the network for 1 day to 1 week before the actual network deployment to obtain the t value for the watchdog algorithm. The one hop delay will be a maximum of two in an exceedingly high traffic network, and in normal traffic it must be less than 1 s. By configuring the t value and start of the network, a sender node enters a watchdog algorithm after forwarding any packet to its parent. If it finds its forwarded packet it will increase the *p*_*j*_ of its parents by one, otherwise it will increase the *n*_*j*_ of its parent by 1. In real human life, slopes that are too steep are detrimental to trust, whereas those that are too gentle foster it. The proposed system follows the same logic by considering +2 in the trust calculation.

### Layer three: decision making

After each trust calculation, the decision-making layer is performed. This layer makes decisions about the parent node using the final trust value. Two procedures are defined in the decision-making layer for each node: a punishment procedure and a forgiveness procedure.

#### Punishment

If node i observes final trust value is less than trust threshold (0.5-PLR) for node j in its neighbor list, it concludes that, blackhole, grayhole or selective forwarding attack occurs, or node j did not forward the packets due to failure, congestion, or selfishness to save energy. In all these cases, the punishment procedure should be considered for node j. The IP address of this node is added to the blocked IPs list (if it is on the list, its flag B is set to one), and a restriction is placed on the preferred parent selection function to avoid selecting node j (and any other nodes that are punished) as the preferred parent. If node j is the preferred parent, another node is selected according to the preferred parent selection criteria. Moreover, the punishments counter for node j( $C_{TF}^j$) is increased by one unit.

#### Forgiveness

Forgiveness procedure is predicted considering the possibility that the punished node may not be the actual attacker node. Sometimes a node behaves negatively because of a temporary situation, such as failure to successfully receive the packets or failure to forward the packets due to hardware error, collision, or lack of resources. Alternatively, the attack might be temporary and the attacker’s access to the node was cut off. The forgiveness procedure for node j starts after the expiration of its punishment time t_f_. If $C_{TF}^j \le TW$, trust of the node is reset to its initial value which is 0.5 and flag B for node j in list of blocked IPs reset to zero. Otherwise, the forgiveness for node j is done with the probability of $\textstyle{1 \over {C_{TF}^j}}$. In other words, for TW (Trust Window) times, initial forgiveness is done, however, if node j repeats the negative behavior and receive punishment again, the probability of forgiveness decreases.

### Layer four: backup and restore

In this research, the backup and recovery layer is introduced for the first time aiming to control a scenario wherein nodes lose their essential information due to an error. After the t_backup_ timeout, the node transmits IP address and the amount of forgiveness for nodes that have been blocked since the previous backup. The root stores this information per node i. Node i reconnects to the network whenever an error occurs, or it is restarted by losing its parent. After choosing a new parent, it will then send a DAO message asking for DIO packets. To distinguish it from DAOs that are typically sent in response to a parent change, we kept a bit flag (B) in that DAO message. Root determines if it is necessary to send the stored data to node i upon receiving this DAO message and inspecting its flag. As a result, it embeds this information in DAO-ACK packets and sends it to node i. Moreover, root stores the attacker IDs by receiving backup message and will alert the network’s nodes about node j if it has received more than k backup messages from different clients identifying the node j as an attacker. All network nodes decrease the priority of node j to be selected as their preferred parent by receiving this information from the root.

### Energy and power consumption

RPLAD3 estimates the node’s battery energy level to decide whether it can perform a specific task, like forwarding a packet. If a node’s battery level is low or its buffer size is almost complete, it may behave differently in a routing decision. Unless they are critical requests for urgent packets, declining emphasizes the significance of the limited resources available to these nodes. Additionally, a node with a high lossy link, limited bandwidth, or a slow processing speed may cause a significant delay in packet delivery. A node with insufficient resources is isolated, given enough time to refill its drained resources, especially battery power, and then given another chance to rejoin the network. Equation (11) (Chu, Horng & Chang, 2019) displays the formula for calculating energy consumption used in the Cooja simulation, and Eq. (12) (Radosavljević & Babić, 2021) displays the formula for calculating power consumption.

(11) $$Energy{\rm \; }Consumption{\rm \; }\left( {mJ} \right) = Energerst\_Value \times Current \times Voltage$$

(12) $$Power{\rm \; }Consumption{\rm \; }\left( {mW} \right) = \displaystyle{{Energy{\rm \; }Consumption{\rm \; }\left( {mJ} \right)} \over {RTIMER\_Second{\rm \; } \times Runtime}}$$

The PDR of RPLAD3 in cooja is calculated by subtracting totalReceived (DATA receive) from totalSent (DATA sent) using the script provided in the Contiki directory’s powertrace.c file. The same script is also used to calculate the end-to-end delay, energy, and power consumption values.

## Results

**Figure 6:**
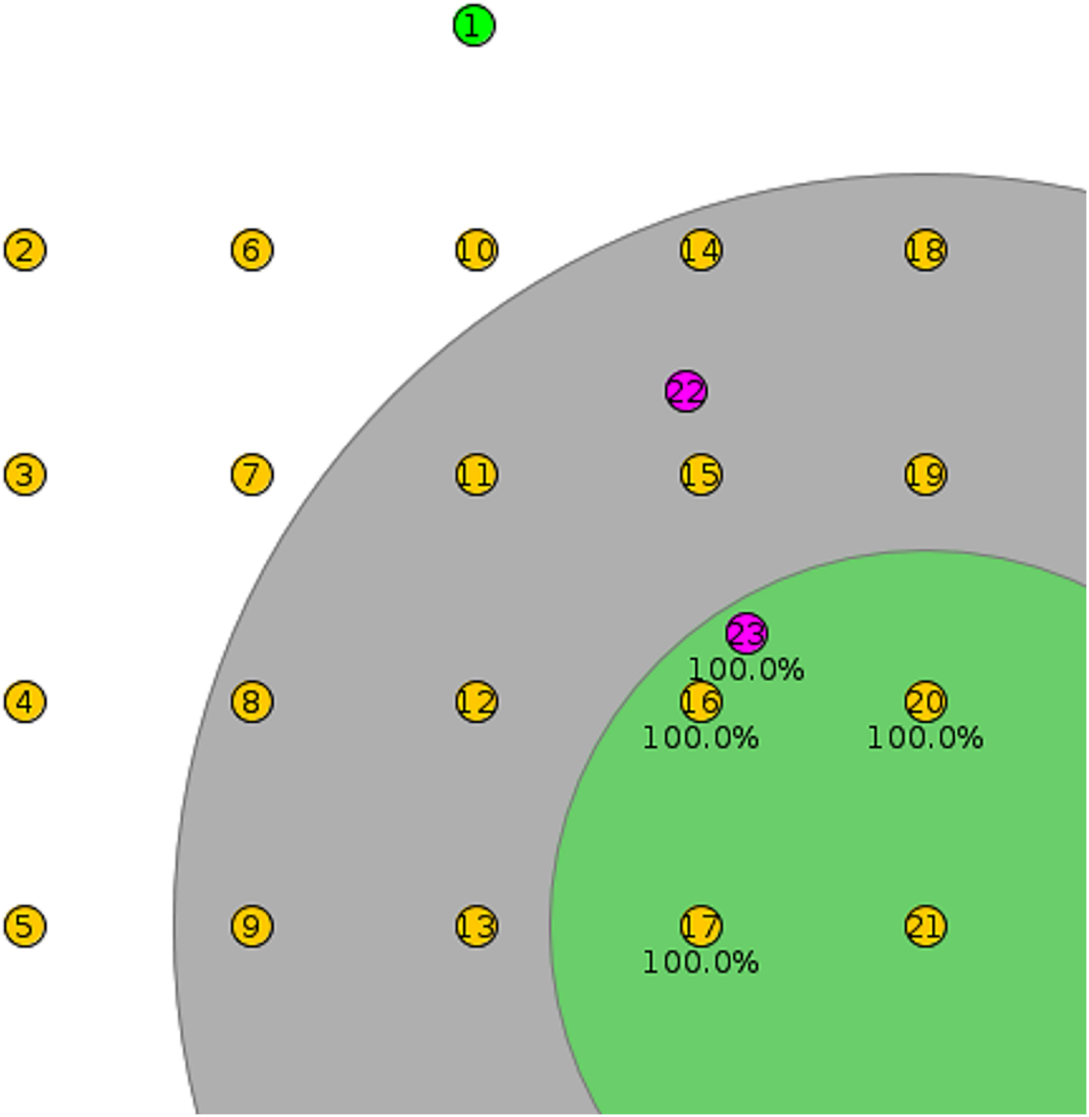
RPLAD3 sample topology in cooja.

As shown in Fig. 6, the root node in each Cooja simulation is in the top middle of the first row, and each client node is arranged into a rectangular structure with five nodes in each row. In Fig. 6, the pink nodes are mobile, while the yellow nodes are client fixed. In each scenario, twenty nodes were used to show the structure of RPLAD3. To show the used structure and mobile nodes more clearly, twenty-two nodes have been added to Fig. 6. Node twenty-one is clicked to view its environment and neighboring nodes.

This study uses the Contiki/Cooja platform to simulate the implementation of RPLAD3 in RPL and analyze the impacts of various threshold values, network sizes, densities, error probabilities, attack rates, and mobile *vs* static frameworks. RPLAD3 is integrated into the RPL protocol during this evaluation process. Table 2 lists all RPLAD3 simulation parameters in cooja. When subjected to similar attacks, the RPL protocol’s performance is evaluated using similar simulation parameters.

**Table 2:** Simulation parameters.

Metrics	To evaluate impact of trust threshold and TW in RPLAD3	To evaluate impact of network size in RPLAD3	To evaluate impact of density in RPLAD3	To evaluate impact of error probability in RPLAD3	To evaluate impact of rate of attackers in RPLAD3	To evaluate impact of mobility in RPLAD3
Number of nodes	20	Variable	20	20	20	20
Distance	30 m	30 m	Variable	30 m	30 m	30 m
Error probability	10%	10%	10%	Variable	10%	10%
Attacker ratio	10%	10%	10%	10%	Variable	10%
Trust threshold	Variable	0.4	0.4	0.4	0.4	0.4
TW	Variable	2	2	2	2	2
Packet type	UDP	UDP	UDP	UDP	UDP	UDP
Packet transfer interval	20 s	20 s	20 s	20 s	20 s	20 s
Packet size	40 bytes	40 bytes	40 bytes	40 bytes	40 bytes	40 bytes
Simulation time	3,600 s	3,600 s	3,600 s	3,600 s	3,600 s	3,600 s
Node type	Skymote	Skymote	Skymote	Skymote	Skymote	Skymote

Four experiments were conducted under different scenarios, and each was based on the average of ten RPLAD3 runs in the Cooja. The experiments are conducted to evaluate the adaptivity, scalability, accuracy, and mobility of RPLAD3. Moreover, an experiment has been done on standard RPL protocol under attacks to compare the results with RPLAD3. The details of the experiments are as follows:

### Experiment 1: RPLAD3 adaptivity evaluation

Using the trust window (TW), a guilty node can be forgiven TW times. Unless the number of attacker flags exceeds TW, a node is not considered an attacker. The false positive rate (FPR) for RPLAD3 attack detection is decreased using this technique. Keep in mind that TW can slightly extend the time it takes to detect an attack. TW value is adaptive to get around this limitation, enabling network administrators to identify the TW value in accordance with network security guidelines. Therefore, trust threshold and TW values could be increased if FPR needs to be reduced, while they could be decreased if detection delay needs to be shortened. The right amount for trust threshold and TW will depend on the network administrator’s security policy, demands, and preferences.

**Figure 7:**
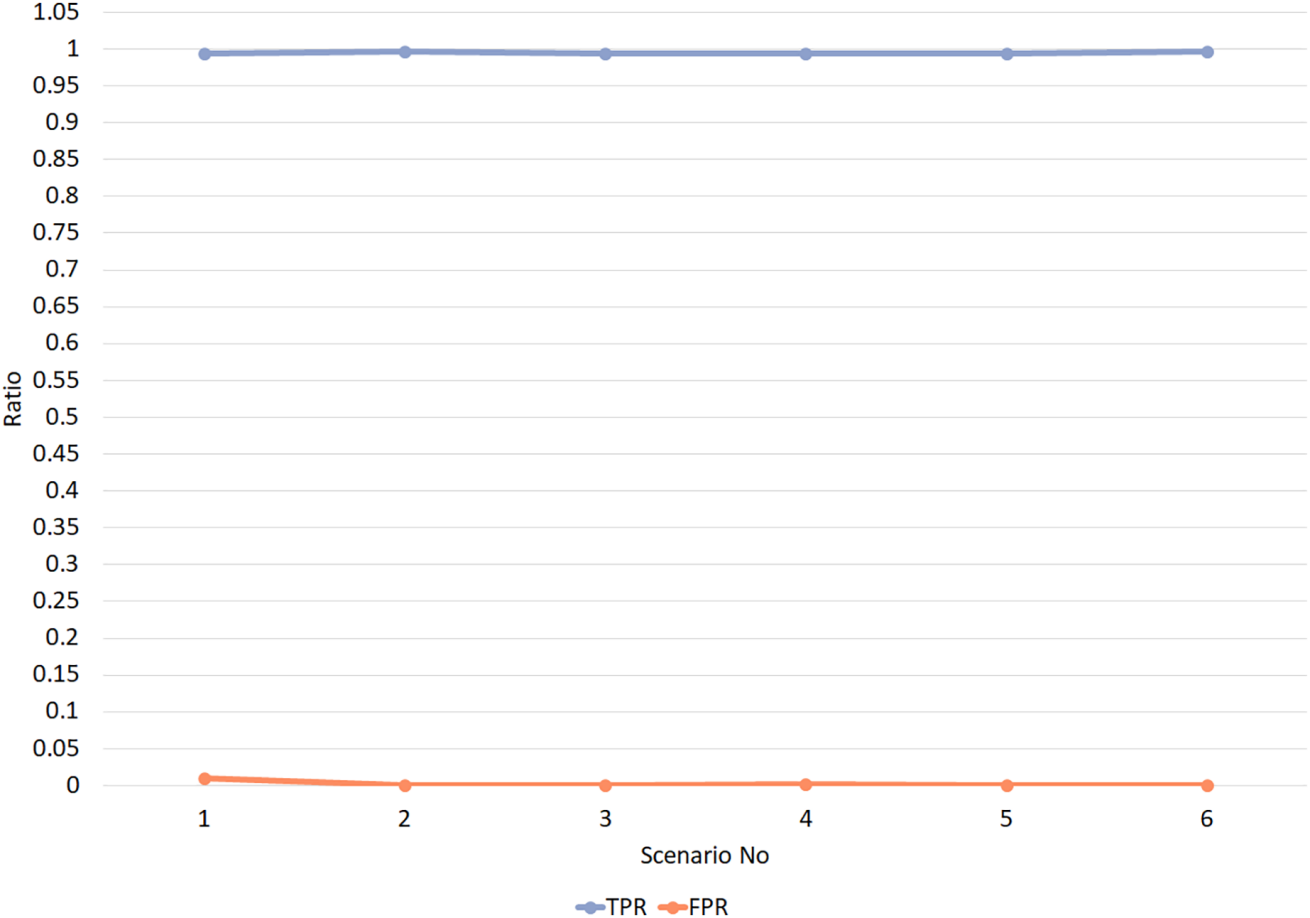
RPLAD3 TPR and FPR with different trust threshold and trust window.

Experiment 1 was conducted in six different scenarios. In scenarios 1 and 2, the value of TW is set to 1, and trust threshold values vary between 0.4 and 0.5. In scenarios 3 and 4, the value of TW is set to 2, and trust threshold values vary between 0.4 and 0.5. Simultaneously, in scenarios 5 and 6, the value of TW is set to 3, and trust threshold values vary between 0.4 and 0.5. TW values vary from 1 to 3 in all scenarios to evaluate the changes in TW values. Besides, trust threshold values are set once as 0.5, based on trust calculation layer 2, and 0.4 other times when the error probability value is subtracted from trust threshold. Figure 7 displays the simulation results of Experiment 1 for different values of TPR and FPR. The evaluation results from experiment 1 undoubtedly reveal extremely high values of TPR, which are almost close to 1, and exceptionally low values of FPR, which are almost close to zero. It is evident that the trust threshold and TW values have no adverse effects on the RPLAD3 trust model. Nevertheless, it significantly improves the network’s performance effectively.

### Experiment 2: RPLAD3 scalability evaluation

Large-scale sensor networks with many sensor nodes require scalable routing attack detection systems to operate effectively. It would be ideal for network performance to remain stable as it grows. The conception and application of such security systems are promised future-generation sensor networks. Experiment 2 is conducted to evaluate the scalability of RPLAD3 and its impact on network size and density while detecting routing attacks. Table 3 displays the results of four distinct Cooja simulator scenarios. RPLAD3 isolated the malicious nodes with low end-to-end delay, FPR, energy, and power consumption after attacks were launched in Cooja. TPR and PDR also experienced rapid growth. We started with ten nodes and increased the nodes in the scenarios stepwise until they reached eighty.

**Table 3:** Experiment 2 results for different network size.

Scenario	# Nodes	PDR	E2E delay	Power	TPR	FPR		Energy
1	10	0.999	0.26	0.92	0.9999	0.000328947		0.552
2	20	0.999	0.41	1.19	0.9917	0.000524061		0.718
3	40	0.998	0.39	1.64	0.9908	0.000380099		0.896
4	80	0.995	0.67	1.67	0.9903	0.000892082		1.002

Since networks must scale from low to high densities to detect malicious nodes in any density, a secure routing system is necessary. RPLAD3 aims to ensure that the sensor nodes can reliably communicate in a wide range of environments while identifying malicious nodes. The correlation between density and node distance is inverse. The density decreases as the distance increases between two nodes and *vice versa*. We have performed three scenarios with node distances ranging from 20 to 40 to evaluate the impact of RPLAD3 on density. In RPLAD3, we evaluated how density impacted PDR, end-to-end delay, TPR, FPR, power, and energy. Table 4 displays the results of the experiment. It can be observed that the FPR is almost close to zero, and PDR and TPR are close to one. Moreover, we experienced low values for end-to-end delay, energy, and power consumption.

**Table 4:** Experiment 2 results for different densities.

Scenario	#Distance (m)	PDR	E2E delay	Power	TPR	FPR	Energy
1	20	0.998	0.39	1.28	0.9918	0.0003153	0.768
2	30	0.999	0.41	1.33	0.9917	0.0006178	0.798
3	40	0.999	0.48	1.43	0.9943	0.000617	0.858

### Experiment 3: RPLAD3 accuracy evaluation

Two different types of experiments are carried out for attacker ratio and error probabilities to evaluate the performance of RPLAD3 regarding detection accuracy. As seen in Table 5, the impact of RPLAD3 on error probabilities is evaluated in four different scenarios with different percentages of error probabilities. The results are evidence that even with a 30% of error probability still, we achieved extremely low values for FPR, end-to-end delay, power, and energy consumption while the values of PDR and TPR are close to one.

**Table 5:** Experiment 3 results for different error probabilities.

Scenario	# Error (%)	PDR	E2E delay	Power	TPR	FPR	Energy
1	0	0.999	0.41	1.31	0.9934	0	0.786
2	10	0.999	0.41	1.33	0.9917	0.000617856	0.798
3	20	0.982	0.45	1.35	0.9811	0.000817	0.81
4	30	0.962	0.52	1.38	0.972	0.0010006	0.828

We have conducted three scenarios to evaluate the impact of RPLAD3 on a different number of attacker nodes. Table 6 reveals the same results for different attacker ratios, which concludes that RPLAD3 can perform accurately even if the number of attackers increases up to 20.

**Table 6:** Experiment 3 results for different number of attackers.

Scenario	Attacker%	PDR	E2E delay	Power	TPR	FPR	Energy
1	0	1	0.34	1.26	0	0	0.756
2	10	0.999	0.41	1.33	0.9917	0.000617856	0.798
3	20	0.968	0.43	1.33	0.9903	0.002088409	0.798

### Experiment 4: RPLAD3 mobility evaluation

The most practical mobility model of the IoT, the random way point mobility, was utilized in the RPLAD3 trust model. It includes options for a mobility framework that defines a route using bond motion techniques. Mobilization is managed as follows: we set the mobile option for a 1-h simulation to travel randomly in a different direction after pausing for 5 min, with a minimum and maximum speed of 1.4 and 5 m/s based on human walking or running. We used the random route from the mobility tools for the experiments with mobile nodes as attackers or victims. The route example given is: (node number, millisecond time, position x, y), indicating the node’s position during that millisecond. Table 7 displays the simulation parameters for the RPLAD3 mobility framework in cooja.

**Table 7:** Simulation parameters for mobility framework in RPLAD3.

Metrics	To evaluate the impact of mobility in RPLAD3
Number of nodes	20
Distance	30 m
Error probability	10%
Attacker ratio	10%
Trust threshold	0.5-PLR = 0.4
TW	2
Framework	Mobile
Packet type	UDP
Packet transfer interval	20 s
Packet size	40 bytes
Simulation time	3600 s
Node type	Skymote
Mobility model	Random way point
Pause time	5 min
Speed	Min: 1.4 m/s Max: 5 m/s (based on the speed of human walk)

**Figure 8:**
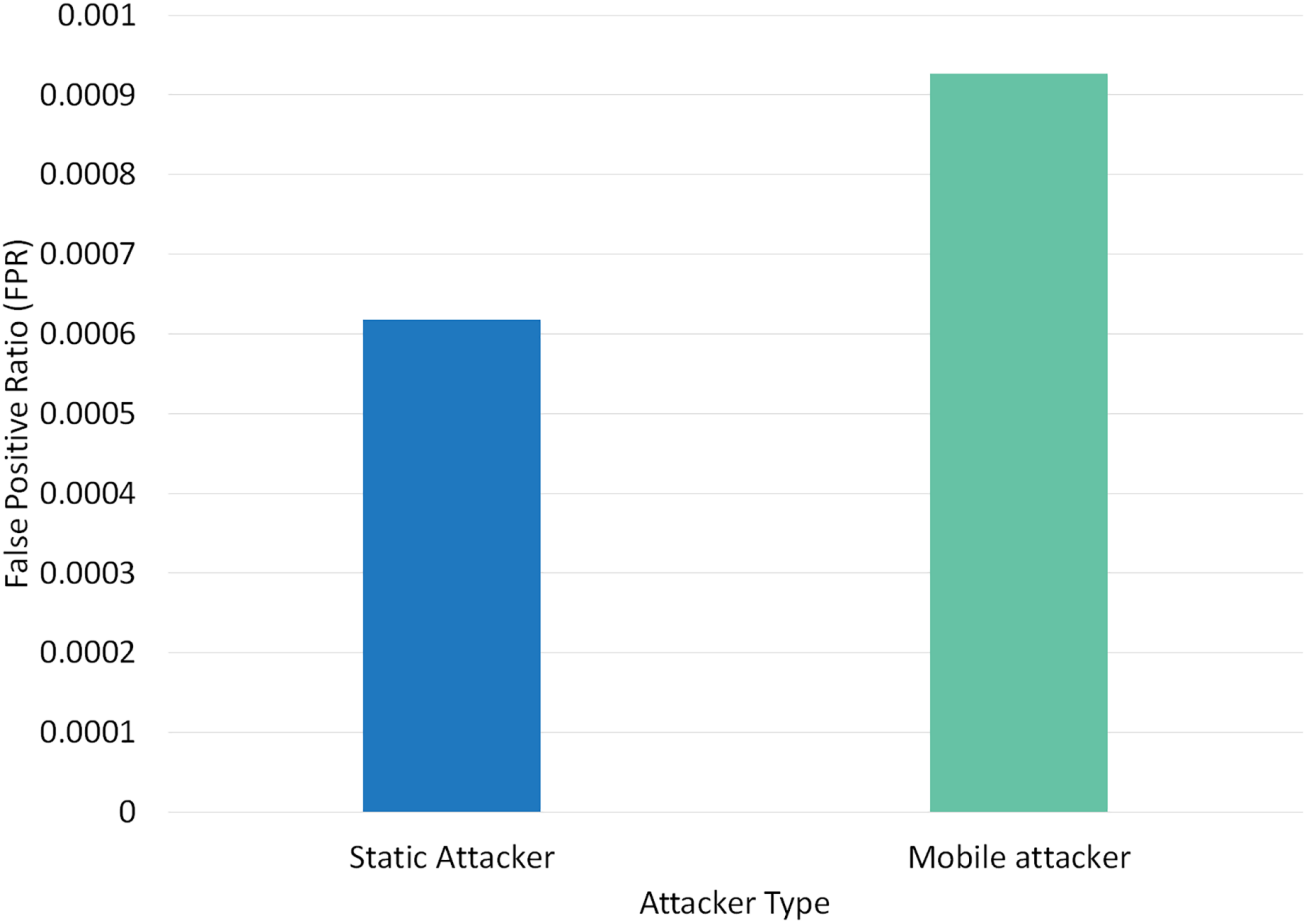
Impact of static *vs* mobile attacker on FPR in RPLAD3.

**Figure 9:**
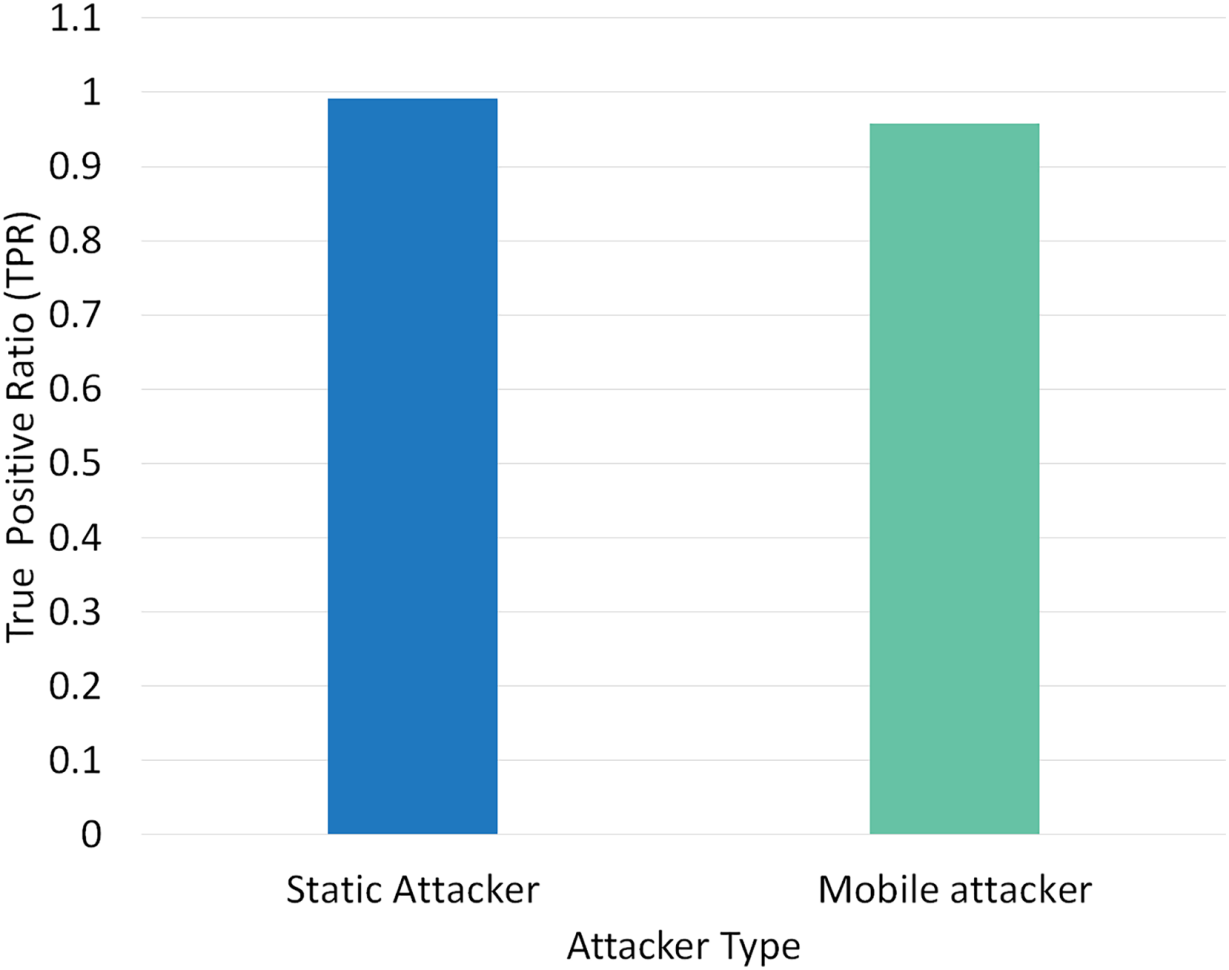
Impact of static *vs* mobile attacker on TPR in RPLAD3.

**Figure 10:**
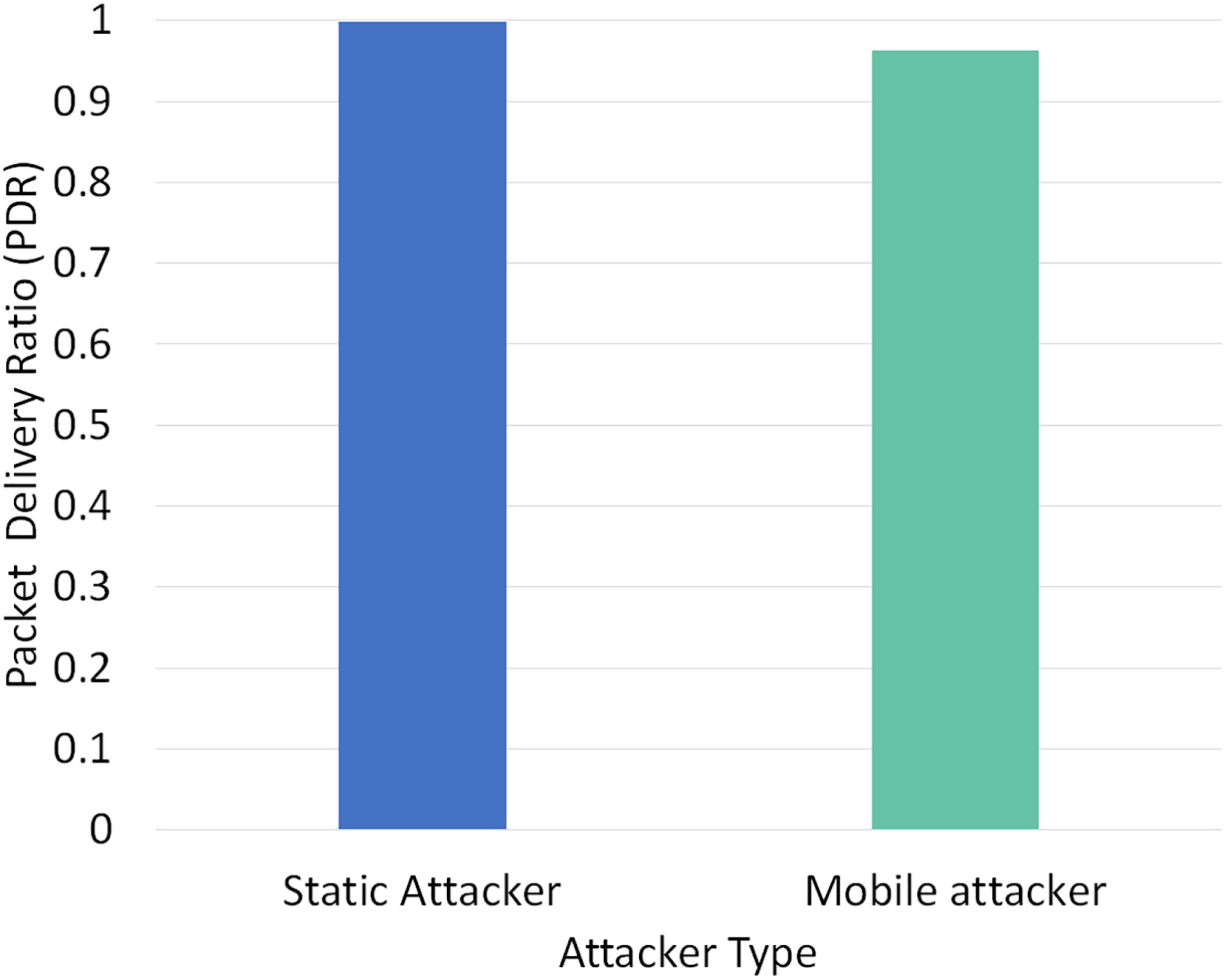
Impact of static *vs* mobile attacker on PDR in RPLAD3.

**Figure 11:**
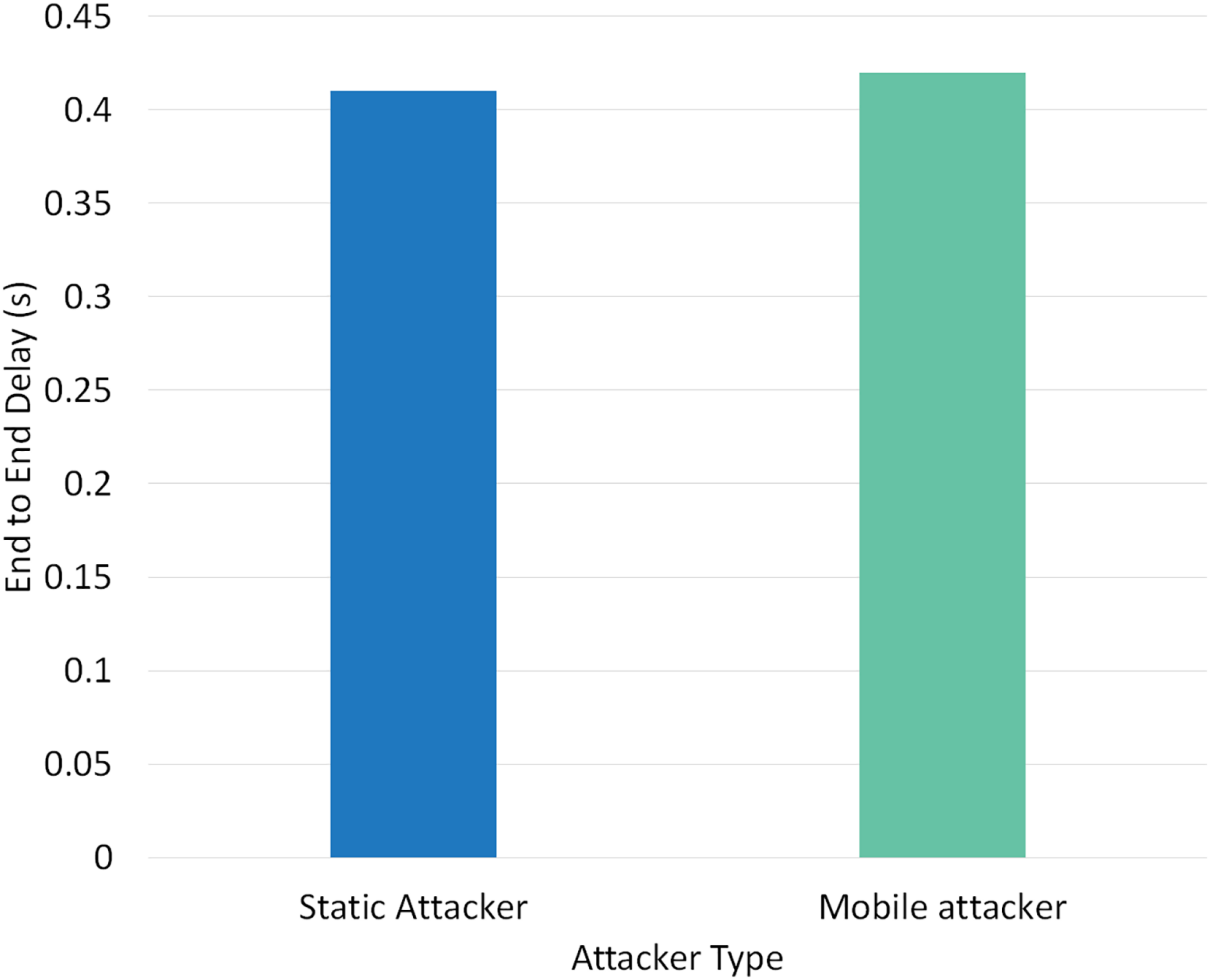
Impact of static *vs* mobile attacker on E2E delay in RPLAD3.

**Figure 12:**
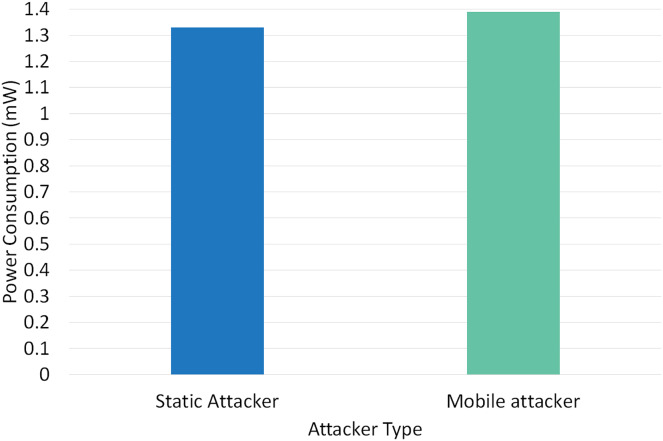
Impact of static *vs* mobile attacker on power consumption in RPLAD3.

**Figure 13:**
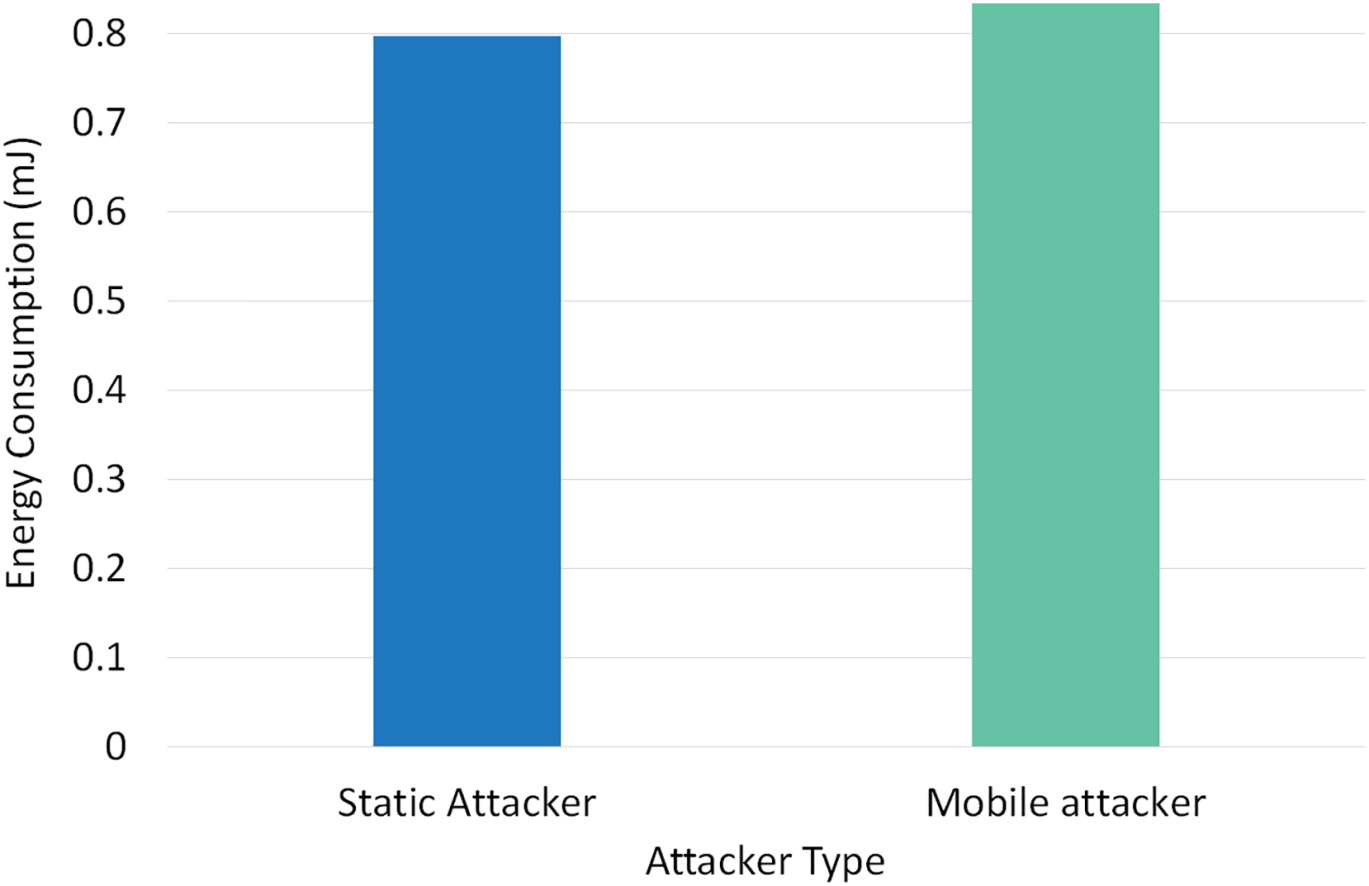
Impact of static *vs* mobile attacker on energy consumption in RPLAD3.

Two different scenarios have been conducted to evaluate the performance of RPLAD3 with a static attacker *vs* a mobile attacker. The results displayed in Table 8 reveal the high performance of RPLAD3, whether it faces a static or mobile attacker. In both scenarios, the end-to-end delay, energy, and power consumption, and FPR are incredibly low, while PDR and TPR are close to one as can be seen from Figs. 8 to 13.

**Table 8:** Experiment 4 results for RPLAD3 static *vs* mobile attacker.

Scenario	Attacker type	PDR	E2E delay	Power	TPR	FPR	Energy
1	Static attacker	0.999	0.41	1.33	0.991735	0.00061	0.798
2	Mobile attacker	0.9633	0.42	1.39	0.957446	0.00092	0.834

Figure 8 depicts the effect of a static *vs* mobile attacker on FPR in RPLAD3. A static attacker’s FPR is almost zero and only 0.0003 less than a mobile attacker. When under attack, the FPR in RPLAD3 performs better than any comparable studies and RPL protocol. Unlike other studies currently available, the proposed method automatically detects and prevents all types of routing attacks with an FPR close to zero.

Figure 9 illustrates the effect of a static *vs* mobile attacker on TPR in RPLAD3. While the mobile attacker only varies from the static attacker in TPR by 0.04, the evaluation results of static attackers are remarkable and almost one. The high TPR in RPLAD3 demonstrates the accuracy and efficacy of the proposed system.

Figure 10 demonstrates the impact of a static *vs* mobile attacker on PDR in RPLAD3. The PDR of a static attacker is almost one, while the PDR of a mobile attacker is only 0.03 higher. When compared to other studies of a similar nature, the proposed system’s PDR and TPR are the highest because they are nearly identical in all experiments and different scenarios, whether from a mobile attacker or a static attacker.

Figure 11 depicts the effect of a static *vs* mobile attacker on end-to-end delay in RPLAD3. The static and mobile attacker evaluation values are 0.41 and 0.42, respectively. The end-to-end delay results of RPLAD3 are satisfactory and slightly lower than previous comparable studies. The proposed system attack detection approach has a minor impact on end-to-end delay, demonstrating the capability of RPLAD3 to identify attacks immediately and with minimal end-to-end delay.

Figure 12 demonstrates the impact of a static *vs* mobile attacker on RPLAD3 power consumption. Both static and mobile attackers consume about the same amount of power to detect attacks, and the evaluation’s findings suggest that power consumption is lower compared to similar studies. RPLAD3 detects and prevents attacks efficiently while using less power, whether the attacker node is mobile or static.

Figure 13 depicts the effect of mobile *vs* static attackers on RPLAD3’s energy consumption. With just a 0.1 variation between static and mobile attackers, RPLAD3’s energy consumption is comparatively deficient. RPLAD3 outperforms other similar approaches in terms of energy consumption, whether confronted with static or mobile attackers.

Three more experiments were conducted to compare the performance of RPLAD3 with a mobile attacker, mobile victim, or both. Table 9 shows the high performance of RPLAD3 with a mobility framework under any attack. FPR is near zero, PDR and TPR are remarkably close to one, while end-to-end delay, energy, and power consumption are low.

**Table 9:** Experiment 4 results for RPLAD3 mobile attacker *vs* mobile victim.

Scenario	Attacker type	PDR	E2E delay	Power	TPR	FPR	Energy
1	Mobile attacker	0.9633	0.42	1.39	0.95744	0.000926	0.834
2	Mobile victim	0.995	0.42	1.42	0.98360	0.001234	0.852
3	Mobile attacker + Mobile victim	0.9512	0.46	1.57	0.90909	0.001543	0.942

## Discussion

RPLAD3 is deployed separately in each node with a complete configuration to be usable in real scenarios with actual nodes. It starts working immediately after the initial state of the network. Each node collects information on their preferred parent and calculates their positive and negative behaviour. Then, this information will go through trust calculation to evaluate the trustworthiness of the parent node. Based on the results of the trust calculation, decisions are made on whether to block the node and label it as an attacker or to trust the node and label it as normal. The backup and restore layer are also utilized in this system for safety and recovery if a node shuts down unexpectedly due to a faulty battery.

The proposed system architecture comprises four interconnected layers: information gathering, trust calculation, decision making, and backup and restore. The four main layers mentioned, which together form the architecture of RPLAD3, were determined based on the literature review findings and a critical evaluation of the research methods and design. Since these layers are connected, each can call the next layer whenever required. Different sublayers are incorporated in each layer which is discussed in detail in the following sections.

Overall, 48 different experiments were conducted to compare the performance of RPLAD3 with standard RPL protocol under blackhole, grayhole, and selective forwarding attacks. The attacks were deployed in the cooja simulator with RPL protocol under the same simulation parameters as Table 2. The number of nodes is 10, 20, and 40, the number of attacker nodes is 1 to 6, the distance between nodes differs from 30 to 40, and the error probability is between 10% and 25%. As can be seen in Table 10, the PDR, end-to-end delay, power, and energy consumption results are obtained in each scenario. A total of 24 scenarios are for standard RPL protocol and 24 for RPLAD3.

**Table 10:** Comparison analysis between the standard RPL protocol and RPLAD3.

					Standard RPL protocol				RPLAD3			
**No.**	**Nodes**	**Attacker nodes**	**Distance**	**Error**	**PDR**	**Delay**	**Power**	**Energy**	**PDR**	**Delay**	**Power**	**Energy**
1	10	1	30	10%	0.46	0.19	0.63	0.378	0.98	0.21	0.88	0.528
2	10	1	30	25%	0.45	0.18	0.66	0.396	0.89	0.21	0.92	0.552
2	10	3	30	10%	0.4	0.11	0.66	0.396	0.94	0.19	0.91	0.546
3	10	3	30	25%	0.38	0.1	0.72	0.432	0.95	0.2	0.95	0.57
4	10	1	40	10%	0.47	0.21	0.79	0.474	0.99	0.29	1.04	0.624
6	10	1	40	25%	0.44	0.18	0.82	0.492	0.97	0.3	1.07	0.642
7	10	3	40	10%	0.41	0.12	0.81	0.486	0.93	0.31	1.06	0.636
8	10	3	40	25%	0.4	0.11	0.89	0.534	0.92	0.38	1.13	0.678
9	20	2	30	10%	0.42	0.25	0.84	0.504	0.93	0.3	1.05	0.63
10	20	2	30	25%	0.39	0.23	0.88	0.528	0.82	0.25	1.1	0.66
11	20	5	30	10%	0.35	0.14	0.86	0.516	0.95	0.3	1.08	0.648
12	20	5	30	25%	0.33	0.12	0.95	0.57	0.92	0.3	1.16	0.696
13	20	2	40	10%	0.44	0.29	1.06	0.636	0.98	0.46	1.28	0.768
14	20	2	40	25%	0.43	0.26	1.08	0.648	0.96	0.55	1.29	0.774
15	20	5	40	10%	0.37	0.23	1.06	0.636	0.95	0.5	1.28	0.768
16	20	5	40	25%	0.3	0.26	1.12	0.672	0.97	0.5	1.34	0.804
17	40	2	30	10%	0.38	0.33	1.08	0.648	0.88	0.35	1.33	0.798
18	40	2	30	25%	0.37	0.29	1.12	0.672	0.86	0.28	1.39	0.834
19	40	6	30	10%	0.33	0.17	1.08	0.648	0.915	0.5	1.35	0.81
20	40	6	30	25%	0.29	0.14	1.16	0.696	0.9	0.33	1.41	0.846
21	40	2	40	10%	0.39	0.27	1.32	0.792	0.96	0.5	1.59	0.954
22	40	2	40	25%	0.37	0.26	1.34	0.804	0.94	0.5	1.61	0.966
23	40	6	40	10%	0.24	0.18	1.33	0.798	0.97	0.6	1.63	0.978
24	40	6	40	25%	0.21	0.15	1.37	0.822	0.95	0.5	1.64	0.984

**Figure 14:**
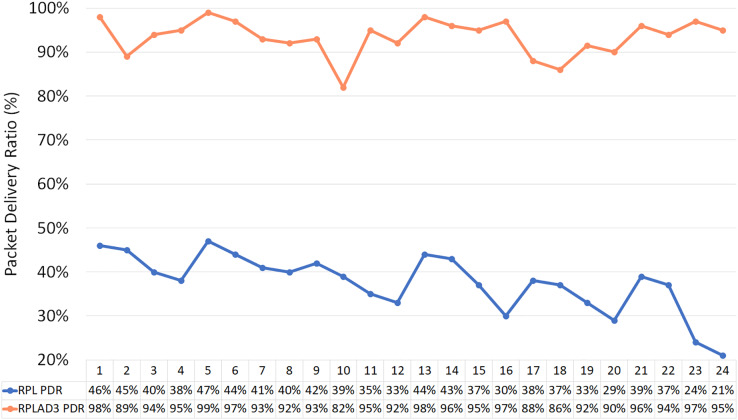
RPLAD3 *vs* standard RPL protocol in terms of accuracy.

Table 11 displays the improvement of RPLAD3 in terms of accuracy against the standard RPL protocol by calculating PDR. The code name for each scenario reveals the simulation setting: N is the total number of nodes, A is the number of attacker nodes, D is the distance between nodes, and E is the error probability. Moreover, Fig. 14 illustrates the accuracy results in which RPLAD3 outperforms standard RPL protocol by the high PDR value, which is almost 100%.

**Table 11:** Comparison of the standard RPL protocol *vs* RPLAD3 in terms of accuracy.

Code	RPL PDR	RPLAD3 PDR	Improvement
N10A1D30E0.1	46%	98%	52%
N10A1D30E0.25	45%	89%	44%
N10A3D30E0.1	40%	94%	54%
N10A3D30E0.25	38%	95%	57%
N10A1D40E0.1	47%	99%	52%
N10A1D40E0.25	44%	97%	53%
N10A3D40E0.1	41%	93%	52%
N10A3D40E0.25	40%	92%	52%
N20A2D30E0.1	42%	93%	51%
N20A2D30E0.25	39%	82%	43%
N20A5D30E0.1	35%	95%	60%
N20A5D30E0.25	33%	92%	59%
N20A2D40E0.1	44%	98%	54%
N20A2D40E0.25	43%	96%	53%
N20A5D40E0.1	37%	95%	58%
N20A5D40E0.25	30%	97%	67%
N40A2D30E0.1	38%	88%	50%
N40A2D30E0.25	37%	86%	49%
N40A6D30E0.1	33%	92%	59%
N40A6D30E0.25	29%	90%	61%
N40A2D40E0.1	39%	96%	57%
N40A2D40E0.25	37%	94%	57%
N40A6D40E0.1	24%	97%	73%
N40A6D40E0.25	21%	95%	74%

**Figure 15:**
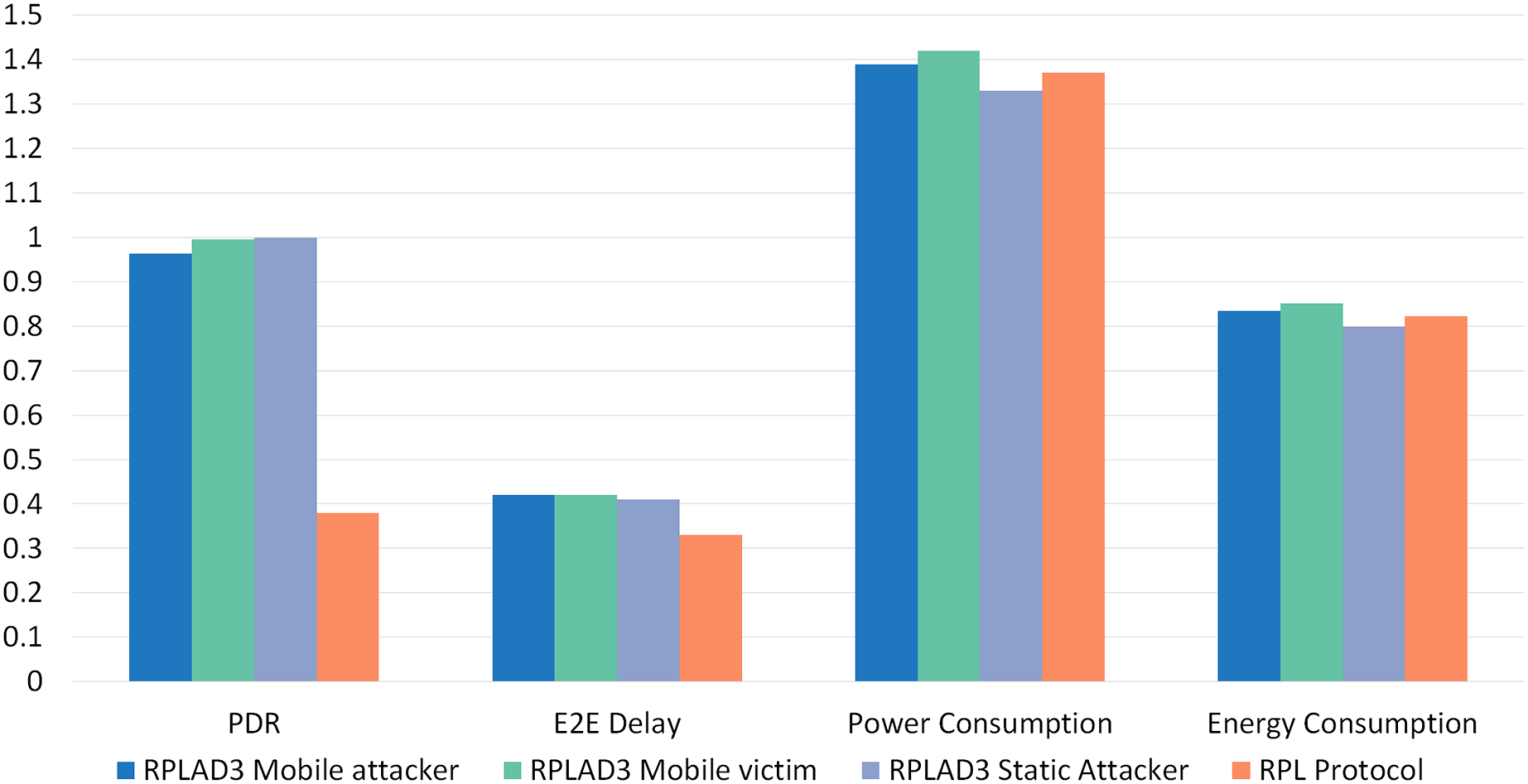
Overall comparison analysis of RPLAD3 *vs* RPL protocol.

Finally, an overall comparison analysis of RPLAD3 *vs* standard RPL protocol is illustrated in Fig. 15, which is evidence of outperforming RPL protocol against RPL protocol by high PDR in all cases and low end-to-end delay, energy, and power consumption.

## Conclusions

RPLAD3 is a trust model routing attack detection system to secure RPL protocol in a distributed IoT. It detects and prevents three routing attacks: grayhole, blackhole, and selective forwarding. RPLAD3 is deployed in each node separately with a complete configuration to be usable in real scenarios with actual nodes. It starts working at once after the initial state of the network. Each node collects information about their preferred parent and calculates their positive and negative behaviour. Then this information will go through trust calculation to evaluate the trustworthiness of the parent node. Decisions are made based on the results of the trust calculation to block the node and consider it an attacker or trust the node and flag it as normal. In this system, backup and restore layer is also utilized for safety and recovery if a node shuts down unexpectedly due to a loose battery. The proposed system assesses two different thresholds, which are adaptive to ensure detection accuracy. The novelty of this system is as per below:

1. It is applicable and resource-constrained in a distributed RPL based IoT as it is lightweight and utilizes a simple framework.
2. It is adaptive and outperforms even in large networks, providing scalable algorithms for trust calculation and isolation of attacker nodes.
3. It detects and isolates malicious nodes and provides a secure route for sensor nodes.
4. It is developed for networks that require secure control and packet exchange with superior levels of availability and integrity.
5. It is compelling and reliable due to its remarkably high detection accuracy, low false positive rate near zero, and incredibly high true positive rate of almost one.
6. It is designed in four interconnected layers to have a full detection capability while detecting attacks.
7. It operates within the mobility framework and can fulfill current industrial desires formed by mobile technologies.
8. It is emulated on sensor nodes with complete setups and routing protocols for all layers, including the physical layer, to be capable of operating with actual sensor nodes.

RPLAD3 examines traffic and node behavior to determine whether nodes behave negatively in the network. Each suspicious node is monitored in the decision-making layer. Even if the nodes are not malicious and are just trying to conserve their drained resources, they are still being observed. After a monitoring and controlling process of these nodes confirms whether they are malicious or resource drained, a course of action is taken under the RPLAD3 architecture.

In future studies, other RPL routing attacks can be detected using the same architecture presented in this article. Moreover, the overhead caused on the network can be discussed and evaluated using RPLAD3.

## Supplemental Information

10.7717/peerj-cs.1309/supp-1 Supplemental Information 1 Source codes.

10.7717/peerj-cs.1309/supp-2 Supplemental Information 2 Data.

## Additional Information and Declarations

### Data Availability

The following information was supplied regarding data availability: The raw data and code are available in the Supplemental Files.
